# Can welding safely be resumed after implanting an extravascular implantable cardioverter-defibrillator

**DOI:** 10.1016/j.hrcr.2025.09.014

**Published:** 2025-10-09

**Authors:** Hans Römers, Max Liebregts, Vincent van Dijk, Lucas Boersma

**Affiliations:** 1 Department of Cardiology, St. Antonius Hospital, Nieuwegein, The Netherlands; 2 Department of Cardiology, Amsterdam University Medical Centers, University of Amsterdam, Amsterdam, The Netherlands

**Keywords:** Cardiology, Implantable cardioverter-defibrillator, Extravascular defibrillator, Welding, EMI, Safety

## Introduction

**Figure 1:**
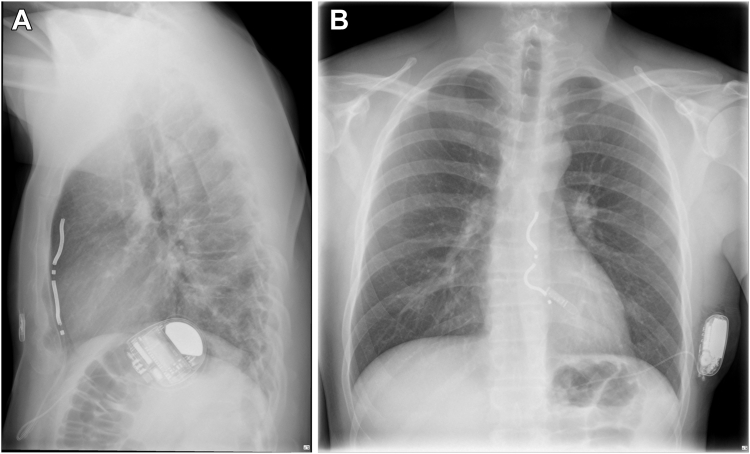
Radiograph after implant position lead and device of an Aurora extravascular implantable cardioverter-defibrillator in anteroposterior and lateral positions.

Since early 2024, the Medtronic Aurora extravascular implantable cardioverter-defibrillator (EV-ICD) has become available for patients who require protection against the consequences of potential ventricular arrhythmias. However, this development also raises new questions for which no definitive guidelines or consensus currently exists. In this case report, we demonstrate that it may be necessary to perform on-site testing in the patient’s daily environment to avoid a false sense of security and ensure safety. This patient, who works as a welder in an automotive workshop, expressed a desire to resume his regular occupational activities but was uncertain whether the equipment he uses would be safe in the context of his device. Welding equipment, owing to its high-power output, has the potential to generate electromagnetic interference (EMI).^1^ EMI is a well-documented concern that may result in inappropriate delivery of therapy or the inhibition of therapy that could be clinically necessary. The Aurora EV-ICD differs from conventional transvenous ICD systems in both lead and device placement, with the lead positioned substernally and the device implanted subcutaneously in the left thoracic wall (Figure 1). In addition, its sensing methodology differs from that of standard transvenous systems.^2^^,^^3^

## Case report

**Figure 2:**
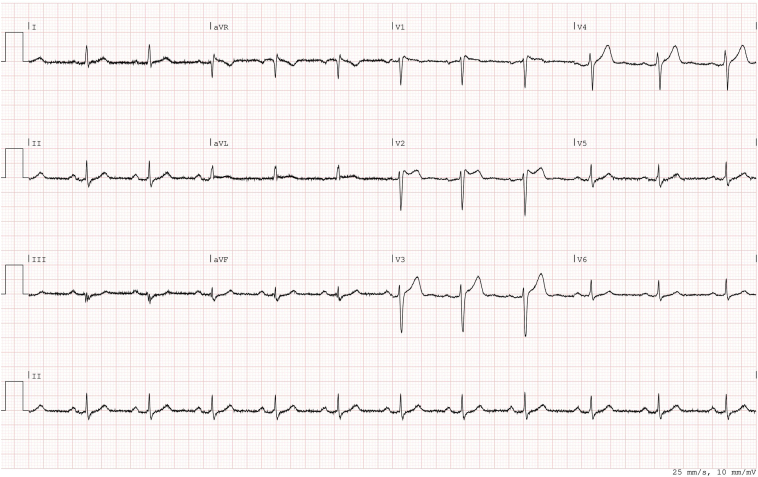
Electrocardiogram.

A 43-year-old man, who had been diagnosed as having Brugada syndrome type 2 (Figure 2) with nonsustained ventricular tachycardias, a positive Ajmaline provocation test, and syncope, received an Aurora EV-ICD implant for primary prevention. Patient has no medication of any kind. The Aurora EV-ICD was programmed with a ventricular fibrillation zone (>200 beats per minute [bpm]), a fast ventricular tachycardia zone (via ventricular fibrillation of 200–240 bpm), and a monitor zone (167–200 bpm).

Months after the procedure, he was preparing to return to work in an auto repair shop. For a while, welding, which is a major aspect of his work, had been performed by his coworkers under his supervision, but he now expressed the desire to resume this task independently. His safety concerns regarding welding were addressed in a consultation at the hospital with both his cardiologist and device technician. Owing to the limited experience with the newly introduced Aurora EV-ICD, this issue was also discussed with a representative from Medtronic. Medtronic had established basic guidelines for welding practice, derived from previous experience with transvenous defibrillator implants.^1^ These guidelines were intended to help prevent unnecessary inappropriate therapy and offer recommendations for the safe use of welding equipment. They included certain limitations regarding the type and output of the equipment used; however, in this case, the welding tools used were more powerful than those recommended by the guidelines. Therefore, the only safe approach was to conduct on-site measurements during the welding process.

4 different welding machines were tested, and each was set up at maximum output to ensure the ultimate potential for detection of EMI (Table 1). Sensing was programmed to maximum sensitivity.

**Table 1:** Welding equipment with maximum power and current

Welding equipment	Maximum power	Maximum current
Spot welding (arc welding)	115 kV	9 kA
Mig welding	4.5 kW	64 A
Induction welding	2000 W	10 A
Spotter	16 kVA	2.8 kA

No modifications were made to the device settings during testing, aside from deactivating therapy to avoid the risk of inappropriate intervention. Electrogram (EGM) recording was configured accordingly: (1) the marker channel reflecting the ring 1 to ring 2 circuit, (2) leadless electrocardiogram capturing ring 2 to can, and (3) EGM 2 monitoring coil 2 to can. These configurations allowed for full tracing acquisition. The sensing vector was only altered to ring 1 to can exclusively upon EMI detection to confirm the device’s sensing of EMI.

**Figure 3:**
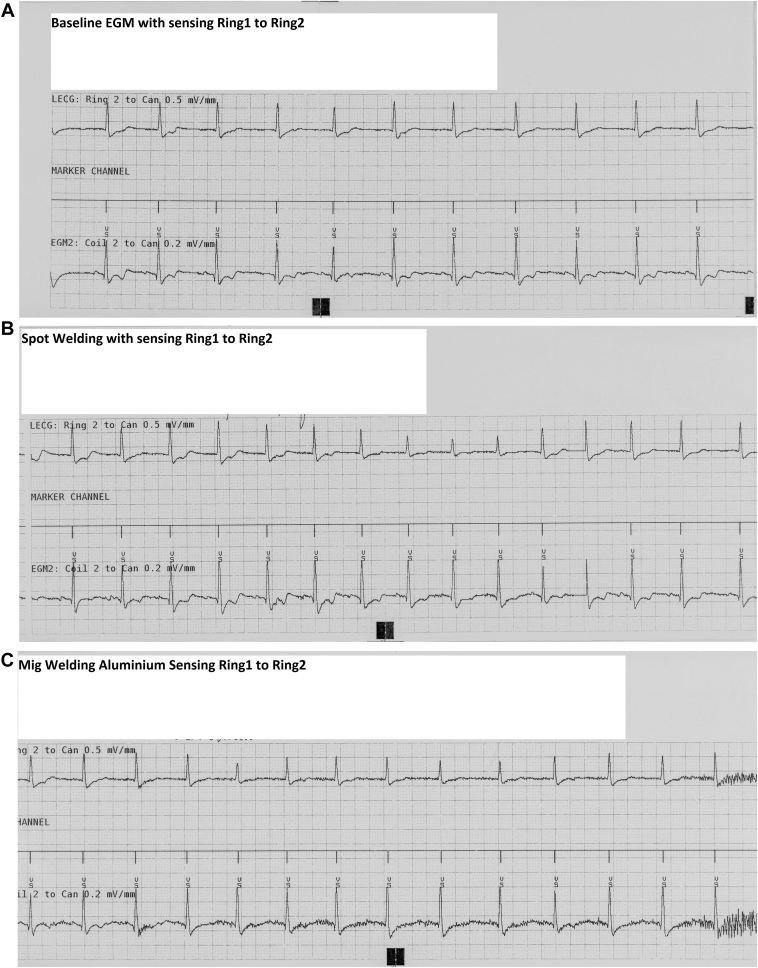

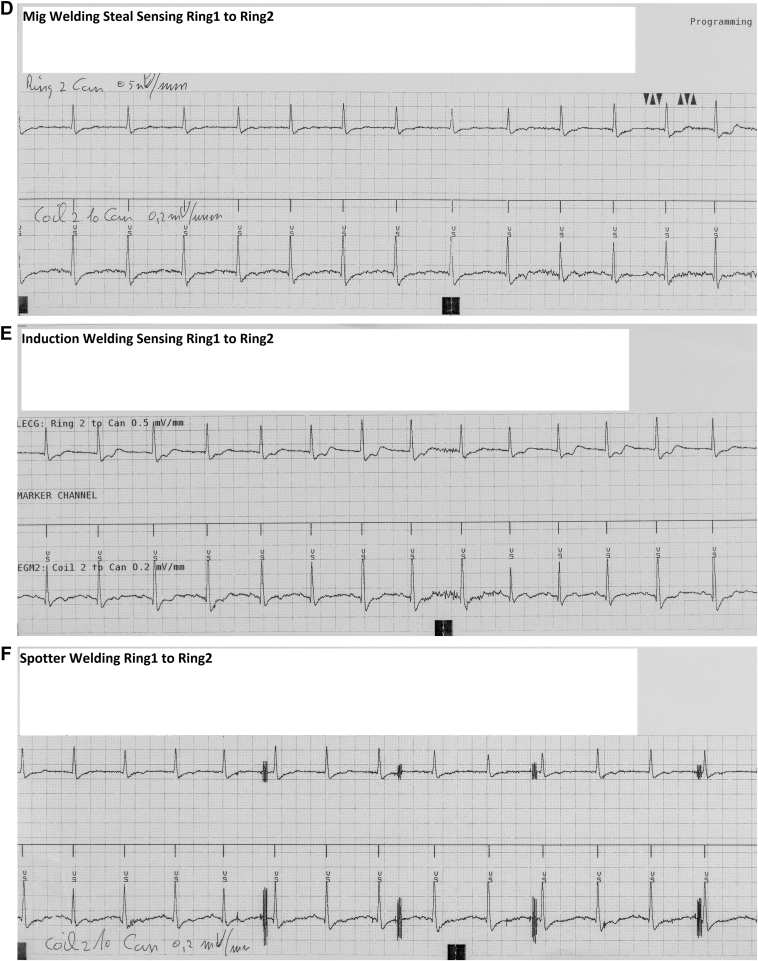

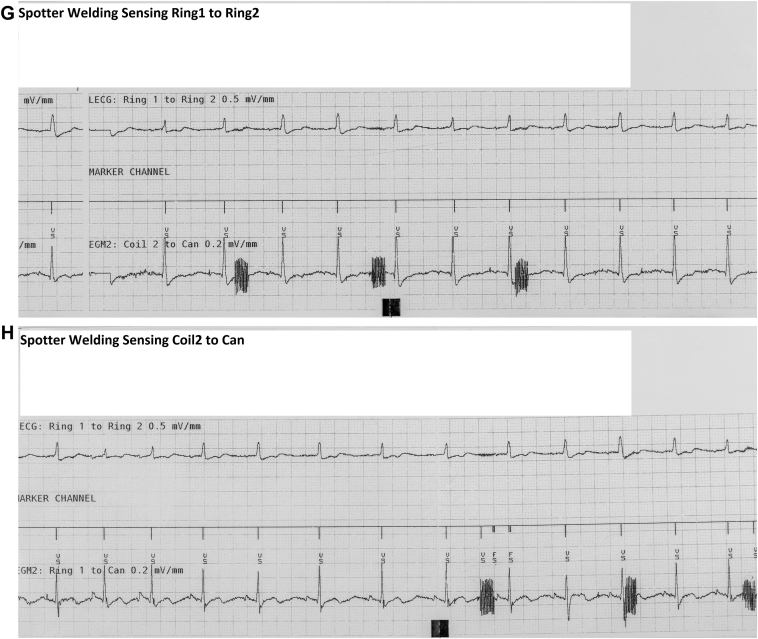
**A:** Baseline with sensing ring 1 to ring 2. Sensitivity 0.15 mV. **B:** Spot welding, no EMI visible. **C:** Mig welding of aluminum. Minimal EMI visible but no detection. **D:** Mig welding of steel, no EMI visible. **E:** Induction welding, minimal EMI visible but no detection. **F:** Spotter welding sensing ring 1 to ring 2, EMI visible on EGM but no detection. **G:** Spotter welding Legm changed to ring 1 to ring 2 with no EMI on the sensing channel. **H:** Spotter welding sensing set to Ring 1 to can, clear detection of EMI. EGM = electrogram; EMI = electromagnetic interference.

Figure 3B–3E demonstrate no or only minimal presence of EMI on the coil 2 to can EGM, with no corresponding EMI detection observed in the ring 1 to ring 2 sensing configuration. Conversely, in Figure 3F, during spot welding, EMI was clearly visible on both the ring 2 to can and coil 2 to can EGMs. However, no markers indicative of EMI were present in the marker channel representing the ring 1 to ring 2 sensing vector. Figure 3G demonstrates that, when the sensing channel was configured as the leadless electrocardiogram, no EMI was detected on the sensing channel. As presented in Figure 3H, the adjustment of sensing to coil 2 to can and the corresponding EGM to ring 1 to can allowed for the clear identification of transient EMI events occurring during welding.

## Discussion

The Aurora system, owing to its specific design and positioning of the lead beneath the sternum, operates with different sensing polarity and sensitivity settings compared with conventional transvenous systems.^2^^,^^3^ The sensing polarity can be programmed in 3 distinct configurations, depending on the quality of the sensed ventricular signals. 2 of the 3 sensing configurations use the can as an integral component of the sensing circuit, creating a larger antenna. The sensitivity settings are also more sensitive than transvenous systems (0.15 mV vs 0.3 mV). This combination may lead to an enhanced susceptibility to noise or EMI. Although EMI has been extensively described in the literature for various contexts, publications specifically addressing EMI related to welding remain scarce.^4^, ^5^, ^6^ The sensing of EMI may trigger inappropriate therapeutic responses from the EV-ICD, thereby representing a clinical risk for the patient. To provide additional safety, the patient was enrolled in remote monitoring so that, if any events occurred, the necessary measures could be taken immediately. Until now, no events have arisen that warrant such intervention.

Occupational activities involving the Aurora EV-ICD are feasible in selected cases; however, they are not universally applicable to all patients. On-site testing is essential to assess electromagnetic compatibility and ensure the safety of both the device and the patient. Therefore, tailored approaches are essential to ensure safety.

## Conclusion

This case report underscores that welding procedures could be conducted safely after real-world environment testing. It was essential to optimize device settings based on patient-specific requirements to ensure protection against potential ventricular arrhythmias without EMI detection. After testing, the definitive device settings were the following:

1. Sense polarity ring 1 to ring 2
2. Blank after sense of 150 ms
3. Sensing threshold decay delay of 360 ms
4. Sensing threshold drop time of 1500 ms
5. Blank after a pace of 250 ms
6. Oversensing prevention medium of 3

On-site testing enabled the evaluation and adjustment of these settings, thereby facilitating safe welding practices for this patient in his daily work environment. For safety assurance, testing should always be tailored to each patient and performed in the actual clinical or occupational setting given that results cannot be generalized across all patients. Incorporating remote monitoring facilitates convenient follow-up and enables prompt identification of potential complications.

## Declaration of generative AI and AI-assisted technologies in the writing process

During the preparation of this work, the authors used ChatGPT 4.0 to improve readability and language. After using this tool, the authors reviewed and edited the content as needed and take full responsibility for the content of the publication.

**Key Teaching Points**

• New medical devices incorporate distinct features that offer novel perspectives on their functionality. A thorough understanding of these features is essential for addressing questions related to electromagnetic interference.
• Collaborating with manufacturing personnel during on-site testing is highly beneficial for ensuring the proper functioning and safety of the medical device.
• Different device settings may respond variably to external signals; therefore, testing should always be conducted in the patient’s actual environment to ensure optimal configuration and safety.
• Testing data may vary among patients and should not be considered interchangeable. Device settings must always be individually tailored to each patient.

## Disclosures

L. Boersma is a consultant for Medtronic, Boston Scientific, Philips, ZOLL, Biosense Webster, and Abbott. V. van Dijk is a consultant for Boston Scientific and Philips. All other authors have no conflicts of interest to disclose.

## References

[1] Can someone with a heart device weld? Medtronic. http://www.medtronic.com/en-us/heart-device-answers/search-results/search-result.can-someone-with-a-heart-device-weld.html.

[2] Swerdlow C., Gillberg J., Boersma L.V.A. (2024). Extravascular implantable cardioverter-defibrillator sensing and detection in a large global population. JACC Clin Electrophysiol.

[3] Swerdlow C.D., Zhang X., Sawchuk R. (2021). Design and preliminary results of sensing and detection for an extravascular implantable cardioverter-defibrillator. JACC Clin Electrophysiol.

[4] Fetter J., Benditt D., Stanton M. (1996). Electromagnetic interference from welding and motors on implantable cardioverter-defibrillators as tested in the electrically hostile work site. J Am Coll Cardiol.

[5] Gurevitz O., Fogel R.I., Herner M.E. (2003). Patients with an ICD can safely resume work in industrial facilities following simple screening for electromagnetic interference. Pacing Clin Electrophysiol.

[6] Tiikkaja M., Aro A.L., Alanko T. (2013). Electromagnetic interference with cardiac pacemakers and implantable cardioverter-defibrillators from low-frequency electromagnetic fields in vivo. Europace.

